# Functional magnetic resonance imaging during emotion recognition in social anxiety disorder: an activation likelihood meta-analysis

**DOI:** 10.3389/fnhum.2012.00347

**Published:** 2013-01-17

**Authors:** Coenraad J. Hattingh, J. Ipser, S. A. Tromp, S. Syal, C. Lochner, S. J. Brooks, D. J. Stein

**Affiliations:** ^1^Department of Psychiatry and Mental Health, Faculty of Health Sciences, University of Cape Town, Cape TownWestern Cape, South Africa; ^2^Department of Psychiatry, Faculty of Health Sciences, University of Stellenbosch, Cape TownWestern Cape, South Africa

**Keywords:** ALE, social anxiety, generalized social phobia, SAD, meta-analysis, fMRI

## Abstract

**Background:** Social anxiety disorder (SAD) is characterized by abnormal fear and anxiety in social situations. Functional magnetic resonance imaging (fMRI) is a brain imaging technique that can be used to demonstrate neural activation to emotionally salient stimuli. However, no attempt has yet been made to statistically collate fMRI studies of brain activation, using the activation likelihood-estimate (ALE) technique, in response to emotion recognition tasks in individuals with SAD. **Methods:** A systematic search of fMRI studies of neural responses to socially emotive cues in SAD was undertaken. ALE meta-analysis, a voxel-based meta-analytic technique, was used to estimate the most significant activations during emotional recognition. **Results:** Seven studies were eligible for inclusion in the meta-analysis, constituting a total of 91 subjects with SAD, and 93 healthy controls. The most significant areas of activation during emotional vs. neutral stimuli in individuals with SAD compared to controls were: bilateral amygdala, left medial temporal lobe encompassing the entorhinal cortex, left medial aspect of the inferior temporal lobe encompassing perirhinal cortex and parahippocampus, right anterior cingulate, right globus pallidus, and distal tip of right postcentral gyrus. **Conclusion:** The results are consistent with neuroanatomic models of the role of the amygdala in fear conditioning, and the importance of the limbic circuitry in mediating anxiety symptoms.

## Introduction

Social anxiety disorder (SAD), or generalized social phobia (GSP), the more pervasive and severe subtype of SAD, are characterized by fear and anxiety in social situations, with individuals experiencing significant concerns about feeling embarrassed or publicly humiliated (American Psychiatric Association, 2000). SAD is a highly prevalent psychiatric disorder, which is characterized by significant chronicity and morbidity (Stein and Stein, 2008). Symptoms often begin during adolescence, with 80% of cases occurring before the age of 18 years (Otto et al., 2001) and if left untreated, are frequently accompanied in later life by depression, substance use disorders, and other anxiety disorders (Watson et al., 2011).

Given the availability of functional imaging techniques, a growing literature on the neural circuitry of SAD has developed (e.g., Freitas-Ferrari et al., 2010). This has led to neurobiological models of SAD implicating a limbic-medial prefrontal circuit in terms of cognitive bias for heightened saliency of negative emotional information (for reviews, see Etkin, 2010, 2012). Specifically, social anxiety in SAD may be mediated by hyperactivation of limbic regions, such as the amygdalae and anterior cingulate cortex (ACC), paralimbic regions such as the hippocampus and insular cortex, combined with aberrant top-down cognitive control of arousal and anticipated fear/perceived threat, especially in relation to the self as represented in medial prefrontal cortex (PFC).

Previous meta-analyses confirm that the amygdala is highly responsive to explicit (Costafreda et al., 2008) and subliminal (Brooks et al., 2012) emotional stimuli, particularly human faces. Moreover, previous meta-analyses and systematic reviews of SAD concur that the amygdalae, as well as the insular cortex are hyper-active in response to emotional stimuli, compared to specific phobia, post-traumatic stress disorder (PTSD), and healthy controls (Etkin and Wager, 2007; Freitas-Ferrari et al., 2010). This is consistent with imaging studies of fear in healthy subjects, which observe that the amygdala and insula are activated in fear paradigms across 55 PET and functional magnetic resonance imaging (fMRI) studies (though the insula, as well as the anterior cingulate, appeared to be more selectively engaged by cognitive aspects of the tasks employed) (Phan et al., 2002).

The aim of this study was to conduct a meta-analysis of activation coordinates extracted from fMRI studies of individuals with SAD that used an emotion recognition paradigm. The meta-analysis was conducted using activation likelihood estimation (ALE), a voxel-based meta-analytic technique that allows one to generate parametric maps of consistent activation across different imaging studies (Laird et al., 2005). We hypothesised that, when passively responding to emotional (vs. neutral) stimuli, individuals with social anxiety (compared to controls) have greater activation in regions associated with emotion regulation, such as the amygdala, insular cortex, basal ganglia, and ACC.

## Methodology

### Definition of social anxiety

SAD is characterized by marked and persistent fear of social or performance situations in which embarrassment may occur, while GSP is characterized when the fears are related to most social situations (e.g., initiating or maintaining conversations, participating in small groups, dating, speaking to authority figures, and attending parties). Both encompass symptoms of being negatively evaluated in social situations, particularly in relation to self-performance (Stein and Stein, 2008). This fear of public scrutiny often leads to avoidance of social situations and biases in social and emotional information processing, particularly of facial expressions (Ekman, 2003), and comorbidities such as anxiety, depression, and substance abuse (Kaufman and Charney, 2000; Kessler et al., 2005). In all studies included in this review, SAD participants were diagnosed according to standardized DSM IV-TR criteria.

## Study identification

Potentially eligible studies that examined individuals with SAD were identified through conducting a search of PubMed, using a combination of the following terms: fMRI, functional magnetic resonance, social anxiety, and social phobia. Study inclusion was restricted to whole-brain (and not region of interest) fMRI studies of emotion recognition in which healthy controls were compared with participants diagnosed with SAD or GSP according to standardized diagnostic criteria as described above.

## Selection process

The selection process took place in three stages. Two independent reviewers firstly assessed the titles and retrieved articles for relevance. Second, the articles that remained eligible were assessed based on the abstract to determine whether any inclusion criteria were not met. The full text of all remaining articles was then assessed using with a data extraction template, constructed for the purpose of organizing and extracting information from included articles. Following this procedure, 244 initial publications were found, which were reduced to 44 after examining the title and abstract. A total of 7 out of 44 studies fulfilled all inclusion criteria, resulting in a combined sample of 91 subjects with SAD and 93 healthy controls.

## Data synthesis and activation likelihood-estimation (ALE)

ALE was conducted using GingerALE software (http://www.brainmap.org/ale/), a contemporary method to analyse brain imaging data. MRICron (REF) was used to illustrate the results. Data from studies reporting Talairach stereotactic (*x*, *y*, *z*) coordinates were extracted and subsequently referred to as foci. We used foci from all studies representing greater activation in cases (vs. controls) to emotional (vs. neutral) stimuli. Studies reporting foci in other atlases (e.g., AFNI, MNI) were converted using the GingerALE convert foci tool. ALE involves the generation of a statistical parametric map (SPM) of brain activation, in this case, in response to a task (emotion recognition), through the quantitative synthesis of whole-brain coordinate data across multiple studies. The likelihood that activation in particular voxels occurs by chance is determined through reference to an empirically derived probabilistic map of brain activity (Turkeltaub et al., 2002; Laird et al., 2005). ALE utilizes voxel-wise resolution of study-level data, avoiding between-study heterogeneity in the positioning of activated voxels via measurement error, which bypasses reliance on publication-specific anatomical labeling (Laird et al., 2005).

For this ALE, the latest version of the ALE software (Eickhoff et al., 2009) was used, with correction using False Discovery Rate (FDR) at *p* = 0.05 and a minimum cluster threshold of 160 mm^3^. Previous versions of GingerALE calculated the probability of voxel activation in the brain on the basis of all foci reported in studies, as if these 3D coordinates were independent of each other. This was problematic, given that those studies reporting a higher number of coordinates (perhaps because they used a lower statistical threshold) would receive more weight with regard to their contribution to the meta-analysis. The latest version of GingerALE (version 2.1.1), on the other hand, identifies each reported coordinate within the study that generated it, by obtaining a single modeled activation (MA) map for each study. The probability that a particular voxel is activated is then calculated as the union of the probabilities for that voxel across studies. This revised algorithm allows weighting for the precision location of the foci reported by each study, based on its sample size (technically, by calculating a study-specific Full Width Half Maximum (FWHM) value which determines how widely-dispersed or “blurred” that activation is). The algorithm assumes that larger studies provide more reliable estimates of activation. The lastest version of GingerALE also restricts voxels of interest to those areas of the brain which have a greater than 10% probability of containing gray matter, as no BOLD signal will typically be observed in white matter.

### Emotion regulation paradigms

Five out of the seven studies included in this meta-analysis presented emotional vs. neutral faces (Phan et al., 2006; Yoon et al., 2007; Evans et al., 2008; Goldin et al., 2009; Klumpp et al., 2010), whereas the remaining 2 studies used emotional statements (Blair et al., 2008, 2011). For example, of the studies presenting faces, participants were required to; (a) rate how happy, sad, and neutral faces looked, after the scan (Evans et al., 2008), (b) to cognitively engage (downplay their emotional response using thoughts, as done in pre-scan trial) or simply look at emotional faces (Goldin et al., 2009), (c) press a button in the scanner to identify type of emotion (positive, negative, neutral) on the face (Phan et al., 2006; Yoon et al., 2007). The 2 studies presenting emotional statements asked participants to think about somebody they know giving a positive, negative, or neutral statement about them or somebody else, and to press a button during the scan when they had read the statement (Blair et al., 2008, 2011). All studies had variations on the emotional theme, but all had in common that emotion recognition occurred.

## Data extraction

The data extracted included; (a) author names, (b) date of publication, (c) journal name, (d) digital object identifier, (e) type of emotion recognition task, (f) subject group numbers, (g) comorbidities and medication, (h) mean age with standard deviation, (i) ratio of gender, and (j) neural activation coordinates. All data included in this meta-analysis are derived from between-subject activation to emotion recognition tasks. In other words, the neutral condition (e.g., neutral faces, statements) is first subtracted by the authors' from the emotion condition (e.g., emotional faces, statements), and the residual activation is compared between cases and controls. The case vs. control comparison is reported as between subject foci in each paper included in this meta-analysis. We illustrate the results using Mango (http://ric.uthscsa.edu/mango/) and MRICron (http://www.mccauslandcenter.sc.edu/mricro/mricron/).

## Results

**Figure 1:**
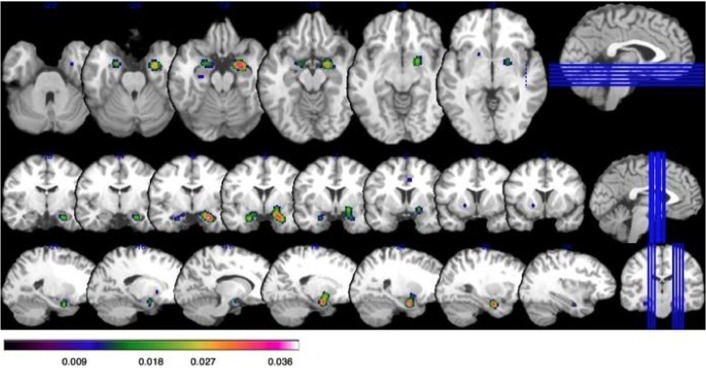
**Increased activation on ALE in those with social anxiety disorder (SAD) at a false discover rate (FDR) corrected threshold of *p* < 0.05, with a minimum cluster size of *K* > 160 mm^3^.** Bar illustrates *z*-scored ALE activation: all coordinates are given as Talairach. **Top row:** axial slices showing activation in the bilateral amygdala (*x* = 26, *y* = −8, *z* = −20; *x* = −22, *y* = −4, *z* = −22); and globus pallidus in the far right slice (*x* = 20, *y* = −2, *z* = −8). **Middle row:** Coronal slices showing activation in the bilateral amygdala and globus pallidus. **Bottom row:** Saggital slices showing activation in the bilateral amygdala and globus pallidus.

**Figure 2:**
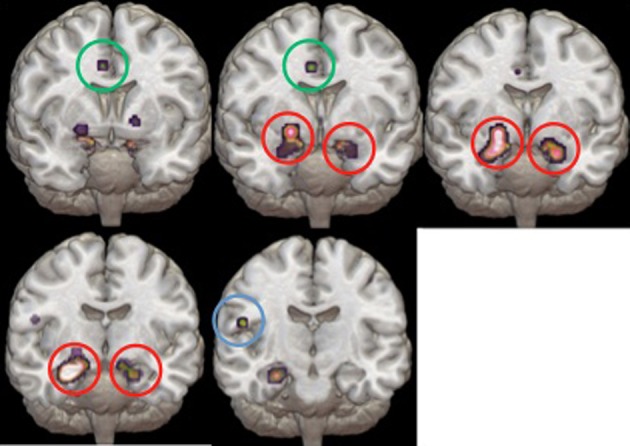
**Volumetric reconstructions of the brain regions most significantly activated in the ALE.** Coronal slices of the bilateral amygdala (circled in red), anterior cingulate cortex (circled in green), and temporal cortex (circled in blue).

Study characteristics and subject demographics are summarized in Table 1. Results from the ALE analysis are summarized in Table 2. Axial, coronal, and sagittal sections of ALE activation areas are shown in Figure 1. Volumetric reconstructions are shown in Figure 2.

**Table 1:** **Characteristics of included studies**.

**Study**				**Sample**				
**Author**	**Title**	**Journal**	**Emotion recognition paradigm/*Brain Atlas***	**Cases**	**Co-morbidity/current medication**	**Age (years)**	**Controls**	**Age (years)**
Blair	Neural response to self- and other referential praise and criticism in generalized social phobia	Arch. Gen. Psychiatry 2008	± vs. neutral statements about self/other; *Tal*	17 (6F; 11M)	None	35.1 (2.47)	17 (9F; 8M)	29.7 (2.28)
Blair	Atypical modulation of medial prefrontal cortex to self-referential comments in generalized social phobia	Psychiatry Res. Neuroimaging 2011	± vs. neutral statements about 1^st^ person or 2^nd^ person; *Tal*	15 (7F; 8M)	None	30.3 (8.49)	15 (6F; 9M)	31.1 (6.37)
Evans	A functional MRI study of amygdala responses to angry schematic faces in social anxiety disorder	Depress. Anxiety 2008	Ratings of happy, sad, vs. neutral faces; *MNI*	11 (7F; 4M)	GAD (*n* = 1); SAx (*n* = 1); MD (*n* = 3) No meds	29.0 (7.5)	11 (7F; 4M)	27.9 (10.6)
Goldin	Neural bases of social anxiety disorder; emotional reactivity and cognitive regulation during social and physical threat	Arch. Gen. Psychiatry 2009	Cognitive linguistic regulation of social (harsh facial expressions) vs. physical (violent scenes); *Tal*	15 (9F; 6M)	None	31.6 (9.7)	17 (9F; 8M)	32.1 (9.3)
Klumpp	Amygdala reactivity to faces at varying intensities of threat in generalized social phobia: an event-related functional MRI study	Psychiatry Res. Neuroimaging 2010	Emotional faces at 3 levels of intensity vs. neutral, button press for valence; *MNI*	12 (GDNR)	GAD (n=2)	28.2 (8.6)	12 (GDNR)	33.6 (9.6)
Phan	Association between amygdala hyperactivity to harsh faces and severity of social anxiety in generalized social phobia	Biol. Psychiatry 2006	Emotional faces vs. pictures of radios; *Tal*	10 (5F; 5M)	None	26.7 (6.8)	10 (5F; 5M)	26.6 (6.8)
Yoon	Amygdala reactivity to emotional faces at high and low intensity in generalized social phobia: a 4-Tesla functional MRI study	Psychiatry Res. Neuroimaging 2007	Emotional faces vs. neutral; *MNI*	11 (6F; 5M)	MD (*n* = 1)	27.0 (6.07)	11 (6F; 5M)	26.9 (6.16)

Tal, Talaraich; MNI, Montreal Neurological Institute; GDNR, Gender differentiation not reported; GAD, Generalized Anxiety Disorder; Sax, Recent history of substance abuse; MD, Major Depression; No meds, Not currently taking medication; None, No meds, no co-morbidity; M, Male; F, Female.

**Table 2:** **Activation clusters: ALE results**.

**Cluster No**	**Volume (mm^3^)**	**Weighted center**			**Extrema value (× 10^2^)**	**Talairach coordinates**			**Activation**	
		***x***	***y***	***z***		***x***	***y***	***z***	**Hemisphere**	**Region**
1	3864	22.55	−5.03	−16.67	3.81	26	−8	−20	Right	Amygdala
					2.3	20	−2	−8	Right	Globus Pallidus
2	1140	−18.91	−4.09	−19.32	2.39	−22	−4	−22	Left	Amygdala
					1.77	−14	−4	−16	Left	Brodmann area 34: medial temporal lobe, amygdala and entorhinal cortex
3	160	−26	−21	−20	1.39	−26	−20	−20	Left	Brodmann area 35: Medial aspect of the inferior temporal lobe: perirhinal cortex of the parahippocampus
4	160	6	3	36	1.39	6	4	36	Right	Brodmann area 24: anterior cingulate
5	160	49.89	−12.1	17.91	1.45	50	−12	18	Right	Brodmann area 43: distal tip of right postcentral gyrus

ALE maps were computed at a false discover rate (FDR) corrected threshold of p < 0.05, with a minimum cluster size of K > 160 mm^3^.

See Table 2 for areas of activation in SAD > HC. Statistically significant increases in activation in response to emotion recognition in individuals with SAD compared with healthy controls was found in the following areas: the right amygdala (*x* = 26, *y* = −8, *z* = −20), left amygdala (*x* = −22, *y* = −4, *z* = −22), left medial temporal lobe encompassing amygdala and entorhinal cortex (Brodmann area 34, *x* = −14, −4, −16), left medial aspect of the inferior temporal lobe encompassing perirhinal cortex and parahippocampus (*x* = −26, *y* = −20, *z* = −20 Brodmann area 35), right anterior cingulate (ACC, Brodmann area 34 *x* = 6, *y* = 4, *z* = 36), right globus pallidus (*x* = 20, *y* = −2, *z* = −8) and distal tip of right postcentral gyrus (*x* = 50, *y* = −12, *z* = 18) (Brodmann, 1909).

## Discussion

For the first time, we present an activation-likelihood meta-analysis of fMRI studies examining the effects of emotion recognition on brain responses in those with SAD. Our most robust finding was that limbic regions were consistently more active in SAD patients than compared with controls in response to emotional stimuli. Specifically, we found significant activation in the bilateral amygdala, left medial temporal lobe, encompassing the entorhinal cortex, left medial aspect of the inferior temporal lobe encompassing perirhinal cortex and parahippocampus, right anterior cingulate, right globus pallidus, and the distal tip of the right postcentral gyrus. It is of note that we found no significant activation of PFC regions, such as the mPFC, which perhaps indicates some competitive interference of arousal brain circuits on higher order functioning, in those with SAD (e.g., for reviews see Etkin, 2010, 2012).

Activation of the bilateral amygdala in response to emotional stimuli was our strongest result. The amygdalae are subcortical gray matter nuclei involved in processing visual emotional cues, particularly signaling fear, aversion, or general salience (Phan et al., 2002). Our data supports previous meta-analyses demonstrating the role of amygdala activation in response to explicit (Costafreda et al., 2008) and subliminal (Brooks et al., 2012) emotional stimuli, particularly in faces. Moreover, previous meta-analyses and systematic reviews of SAD indicate hyperactivity in the amygdalae in response to emotional stimuli (Etkin and Wager, 2007; Freitas-Ferrari et al., 2010). It is of note that the previous meta-analyses of SAD differ from ours in that they did not use the ALE approach, which is a well-validated, systematic, and computational method to objectively to meta-analyse neural activations (Radua et al., 2012). For example, Etkin and Wager (2007) fMRI and PET studies reporting on SAD, specific phobia, PTSD and fear conditioning, and found similar neural activation in these groups. Furthermore, Freitas-Ferrari et al. (2010) conducted a systematic review of fMRI, PET, SPECT, MRS, and structural MRI in studies examining SAD, and reported amygdala and other limbic region activations. The added value of our meta-analysis is that we use ALE to focus only on fMRI studies during emotion recognition in SAD. Thus, our and previous meta-analytic and review data robustly implicate the role of the bilateral amygdala in emotional processing. Specifically, hyperactivation of the amygdalae likely contributes to the mediation of SAD, particularly when viewing emotional information from people's faces.

Animal literature is also concurrent with the observation in this meta-analysis that bilateral hyperactivity in the amygdala occurs when viewing stimuli perceived as emotionally threatening or aversive (de Carvalho et al., 2010). For example, projections from the amygdala to the brainstem contribute to a “quick and dirty” response in animals and humans to potentially salient stimuli, and projections from the amygdala to the cortex simultaneously contribute to the experience of other cognitive aspects of emotional processing (LeDoux, 2003). However, data suggests that the function of the amygdala extends beyond fear processing and is involved in processing of general salience and arousal to both positive and negative emotional processing (Siebert, 2003). Thus, hyper-arousal in limbic brain circuits, regardless of the valence of the emotional stimulus, may underlie the mediation of SAD.

We also observed greater activation in the parahippocampal gyrus (BA34, 35) in those with SAD, consistent with the involvement of this structure in Pavlovian contextual fear conditioning in both animals and humans (Alvarez et al., 2008). Previous systematic reviews and meta-analyses of functional neuroimaging in SAD also noted greater activation in this region (Etkin and Wager, 2007; Freitas-Ferrari et al., 2010). Parahippocampal hyperactivation in SAD has been interpreted as indicative of disruptions to contextual fear conditioning and an inability to assign accurate saliency value to a stimulus. Additionally, increases in gray matter volume in the parahippocampus have been demonstrated in SAD (Talati et al., 2013), indicative of plasticity that may underlie hyperactivity in this region.

The ACC showed greater activation in SAD compared to healthy controls in this meta-analysis. Animal and human studies demonstrate that the ACC plays a key role in the regulation of cognitive and emotional processing (Whalen et al., 1998; Bush et al., 2000), particularly in relation to conflict monitoring/error detection of discrepancies between predicted and actual outcomes (Botvinick and Watanabe, 2007; Yeung and Nieuwenhuis, 2009; Kim et al., 2010). Moreover, the ACC is highly activated during the anticipation of pain (Straube, 2009). Activation of the ACC may therefore point to compensatory efforts to regulate high anxiety states in individuals with SAD, or the anticipation that a socially-painful experience may subsequently ensue. The findings here are again consistent with those of other systematic reviews and meta-analyses in SAD (Etkin and Wager, 2007; Freitas-Ferrari et al., 2010). Finally, alterations in gray matter volumes have recently been associated with anxiety disorders, especially in the ACC region (Radua et al., 2010), however, it is still unclear how structural differences relate to functional aberrations in this region.

We are the first to demonstrate in a meta-analysis that increased activation of the globus pallidus is associated with SAD. The globus pallidus appears to play an important role in motor control (Salih et al., 2009). One possibility therefore is that activation of this area reflects disruption of the motor system as a component of the emotional response in SAD. However, the globus pallidus has also been implicated in affect regulation (Murphy et al., 2003), and in both the processing of information from complex stimuli and in aversive responses to fear and anxiety (Talalaenko et al., 2008). Thus, activation in this region may reflect increased statistical power of this meta-analysis relative to those previously conducted.

Additionally, hyperactivity in the distal tip of the right postcentral gyrus was observed. This area has previously been implicated in conveying pure somatosensory information of an auditory nature (Job et al., 2011). Literature on the functional significance of BA 43 in SAD is lacking, but it seems reasonable to suggest that hyperactivity in this region in people with SAD may be associated with a state of hypervigilance, often in concurrence with anxiety states.

Our data is in line with contemporary neural models of anxiety disorders (for reviews, see Etkin, 2010, 2012). Specifically, a limbic-medial prefrontal circuit is implicated in an aberrant information processing circuit, whereby negative socio-emotional information is given greater salience, due to activation of limbic arousal circuits. Furthermore, the medial prefrontal, self-referential system is perhaps overloaded by bottom-up arousal information, biasing negative emotional stimuli, particularly in relation to the self, such that the value of the external stimulus is heightened, and the value of self-related goals, such as autobiographical memory, self-goals, and experience-based competence are reduced. However, there were a number of regions emphasized in the literature as important components of aberrant brain activity in SAD that were not evident in this meta-analysis. Of particular note, insula activation did not differentiate SAD from healthy controls, despite the recognition of the importance of this structure in mediating anxiety (Craig, 2009; Holzschneider and Mulert, 2011). This may reflect the emphasis on responding to external fearful stimuli rather than to interoceptive signals in the particular paradigms deployed in the included studies (Paulus and Stein, 2006).

There are some limitations to our meta-analysis that must be considered and which may temper the strength of our findings. Firstly, only 7 fMRI studies, with small sample sizes were included. In addition, two of these studies employed a paradigm which did not rely on emotional recognition of facial expressions. Furthermore, due to the small samples, it is difficult to investigate how socio-demographic variables or neurobiological factors (e.g., gene variants) contribute to the brain activation we observe. Nevertheless, this study is the first to conduct an activation-likelihood meta-analysis of fMRI studies examining neural correlates of emotion recognition in SAD. Our findings support a growing body of work on development of a neurocircuitry model of SAD. Future work on larger samples, including perhaps collaborations across different sites, is needed to address more comprehensively some of the likely sources of inter-individual variance (Furmark et al., 2004; Smoller et al., 2008).

In conclusion, our meta-analysis confirms that increased activation of the bilateral amygdala is a prominent feature in those with SAD, particularly in response to socially emotive stimuli. The contribution of other brain regions implicated in neurobiological models of SAD, such as parahippocampus and anterior cingulate, are also highlighted (Morgan et al., 1993). Thus, hyperactivation of limbic circuitry likely mediates the symptoms of SAD, and may be a target for clinical interventions and future research.

### Conflict of interest statement

The authors declare that the research was conducted in the absence of any commercial or financial relationships that could be construed as a potential conflict of interest.
